# The Fourteenth International Congress of Medicine

**Published:** 1901-09

**Authors:** 


### The Fourteenth International Congress of Medicine.

The Fourteenth International Congress of Medicine, Madrid, 1903.—The Fourteenth International Congress of Medicine will be held at Madrid, from the 23rd to the 30th April, 1903, under the patronage of King Alphonso XIII. and the Queen Regent. The subscription is 30 pesetas (equivalent to 24 shillings). Ladies accompanying members will be entitled to the privileges of the members, in the reduction of fares on the Spanish Railways, &c., on payment of 12 pesetas apiece (or 10 shillings).

The National Committee for Great Britain and Ireland, remains the same as at the Paris Congress. Sir William MacCormac, Bart., K.C.B., K.C.V.O., being the President. The Honorary Secretaries are Dr. Percival Horton-Smith, of Upper Brook Street, Grosvenor Square, and Mr. D'Arcy Power, F.R.C.S. Eng., of 10a Chandos Street, Cavendish Square, London, W.

